# A high-frequency dataset of sea level observations from low-cost pressure sensors

**DOI:** 10.1038/s41597-025-05342-9

**Published:** 2025-06-13

**Authors:** Lucia Pineau-Guillou, Pascal Lazure

**Affiliations:** IFREMER, Laboratoire d’Océanographie Physique et Spatiale, UMR 6523 (IFREMER, CNRS, IRD, UBO), IUEM, Brest, France

**Keywords:** Physical oceanography, Physical oceanography

## Abstract

A total of 30 low-cost bottom pressure sensors sampling at 2 Hz were developed and deployed in a nearshore environment during 4.2 months. The experiment took place in Blancs Sablons, a sandy bay near Le Conquet (France), during 2023–2024 winter. All the 30 sensors were successfully recovered. Among them, 22 sensors functioned properly recording full data, 5 sensors partially recorded data due to battery failure or malfunctioning, and only 3 sensors did not work, due to small holes in their plastic protection. Comparison with a commercial pressure gauge revealed the good performance of the low-cost sensor, with a large autonomy, few missing data, and a robust wave-to-wave comparison. However, a small drift of around −0.001 bar/month was detected. Pressure data were converted into sea levels, related to Chart Datum, and validated against permanent instruments (wave buoy and tide gauge) located close to the site. The high-frequency dataset can now be used to validate ocean and wave models, or fine scale altimetry missions such as SWOT (Surface Water and Ocean Topography).

## Background and Summary

In a warming climate, extreme sea levels are increasing^1^. This increase is firstly due to mean sea level rise^2–4^, which reaches 3.1 ± 1.4 mm/yr globally at the end of the 20th century^5^. At the same time, more and more people live in coastal areas^6^. The population is then more exposed to coastal risks, such as nearshore erosion and large flooding, which may have catastrophic consequences. For example, in 1970, Bhola cyclone, one of the worst natural hazard, hit Bangladesh coasts killing 300 000 people^7^. In Europe, the 1953 North Sea Flood caused more than 2 000 deaths^8,9^, whereas more recently, on the 27-28th of February 2010, Xynthia storm severely hit the French Atlantic coasts, causing huge damage and human losses^10–12^. It is then essential to better understand sea level extremes, in order to robustly predict them, and thus protect our coastal areas.

In situ sea level observations are essential, for many reasons. First, they help to understand physical processes^13–17^ and investigate changes^18^. For example, observations reveal how waves and storm surges can significantly contribute to sediment transport^19^ and erosion along the shore line^20^. Second, sea level observations are useful to validate ocean and wave models^11,14^, and improve parameterizations^17,21^. Third, observations are necessary to calibrate and validate satellite altimetry data, and identify possible long-term instrumental drifts^22–24^.

Bottom pressure gauges are commonly used to measure sea level and waves^13–17^, pressure data being converted into water levels^25^. However, commercial instruments may be expensive, preventing from deploying a larger number of sensors, particularly in coastal areas where they can be lost^26^. But the promising capabilities of low-cost sensors^26–28^ allow now to plan larger experiments with more instruments.

A total of 30 low-cost bottom pressure sensors sampling at 2 Hz were developed and deployed in a nearshore environment during 4.2 months. The experiment took place in Blancs Sablons bay, near Le Conquet (France), between 14 December 2023 and 25 April 2024. The sensors are classical low-cost pressure gauge^26–28^, with some particularities: a strong autonomy (more than 4 months at 2 Hz) and an ultra-low cost (less than $100). During the experiment, sensors were fixed on 1-meter-wide duckboards, weighted at all four corners, to prevent the structure from moving. All the 30 sensors were successfully recovered. Among them, 22 sensors functioned properly recording full data, 5 sensors recorded only partial data due to battery failure (4 sensors) or malfunctioning (1 sensor), and 3 sensors did not work, due to some holes in the sensor protecting plastic bag. Pressure data were processed to reconstruct 2 Hz sea levels, following the Karimpour and Chen^25^ method. Sea levels were related to Chart Datum, using the low waters concordance method^29,30^. Comparison with a commercial RBR solo pressure gauge revealed the good performance of the low-cost sensor, with a large autonomy, few missing data and a robust wave-to-wave comparison (Root Mean Square Error of 0.009 bar between the two instruments). However, a small drift of around -0.001 bar/month was detected. The waves and storm surges compared well with permanent instruments, close to the site. Note that sea level data may display quite significant trends (−1.7 cm/month in average), which leads to consider the data with caution for applications requiring a very stable vertical reference.

In this paper, we present a valuable high-frequency sea level dataset, available for the scientific community^31^. Data records consist of 2Hz raw pressure data and sea level data during 2023–2024 winter at 27 positions in Blancs Sablons bay, near Le Conquet, France. This dataset can be used to investigate the wave transformation in a nearshore environment. The dataset can also be used to validate ocean and wave models, or fine-scale altimetry missions such as SWOT (Surface Water and Ocean Topography^32^), whose tracks are partly close to Le Conquet.

## Methods

### Material

**Fig. 1:**
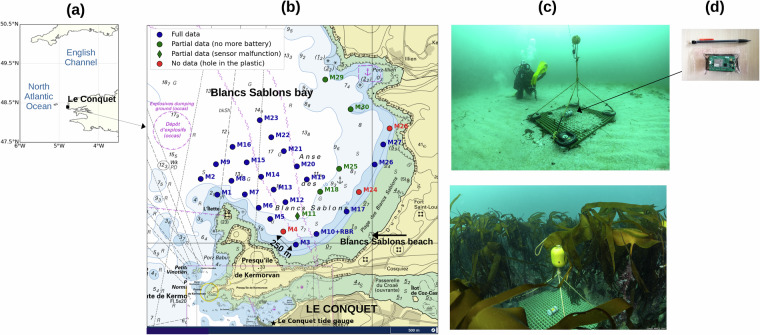
(**a**) Location of the field campaign (**b**) Localisation of the 30 low-cost bottom pressure sensors (**c**) Structures moored at sea, with the sensors placed at the center in a protecting tube © Erwan Amice CNRS (**d**) Low-cost pressure sensor (Mastodon V4 card). The pencil indicates the size of the sensor.

The low-cost sensors, called Mastodon, are jointly developed by Ifremer (France) and a private company (Mahé Informatique Industrielle, France). The Mastodon V4 card (Fig. 1d) is a standalone electronic device designed to measure temperature and pressure. The sensor measures the bottom pressure similarly to other low-cost pressure gauges recently described^26–28^, but its advantages compared to others is a long autonomy (well over 4 months recording at 2 Hz), and a packaging method which ultimately allows for a very cheap instrument (total cost of less than $100 for a production of over 100 units).

The Mastodon V4 card (Fig. 1d) includes a STM32L431 microcontroller for data processing, an ESP32-S2 WiFi module for wireless communication and two sensors: an ADT7320 for temperature and a MS5837 for pressure. The recorded data are stored in a Kioxia TC58CVG2S0HRAIJ flash memory with a capacity of 500 Mo (which can be doubled if necessary), allowing several months of data storage. The card, optimized for low energy consumption, is powered by two AA batteries. Before use, the card must be programmed. The STM32L431 microcontroller is flashed using STLINK V3 with the STM32CubeProgrammer software, while the ESP32-S2 WiFi module is programmed separately using the ESP-PROG tool and Flash Download Tool from Espressif. The programming process involves connecting the card via dedicated connectors and uploading firmware that manages data acquisition, storage, and wireless communication. Once programmed, the card can operate autonomously, collecting and storing measurements according to predefined schedules.

To configure and interact with the Mastodon card, the IHMMAS4 software, developed in C++, provides a user-friendly interface. Users can set measurement parameters, launch data acquisition, and download stored data via WiFi. The card’s WiFi module is only activated when a magnet is placed near the sensor, ensuring energy efficiency. The software displays real-time status, measurement logs, and battery levels, allowing easy monitoring and control of the system, even for non-experts.

To keep the cost of the complete instrument to a minimum, the electronic card and the two AA batteries are inserted in a heat-sealed flexible bag (intended for medical use) filled with Marcol 82 dielectric oil. The advantage of this type of packaging is its low cost. As the pressure is identical inside and outside the bag, the instrument can be immersed to a depth of 300 m (the limit of the pressure sensor) without risk of leakage. Depending on the application, the bag can be protected by a PVC tube through which water can circulate and/or by a fine copper mesh to limit biofouling. However, replacing the batteries after long-term deployment requires repackaging, which takes longer than conventional packaging at atmospheric pressure.

The sensors were deployed on wide and flat structures (Fig. 1c). The structures were duckboards of 1 m long and large, with weights of 5 kg at each corner, to ensure maximum stability during the experiment, and avoid any horizontal or vertical movements. Each structure weighted approximately 36 kg. The sensors, protected by a thin mesh and a PVC tube, were fixed at the center of each duckboard.

To facilitate the recovery of the structures by the divers, a yellow buoy was placed around 1 m above the instrument (see Fig. 1c). For structures moored in deep waters (between 5 and 20 m), a secondary buoy was added in subsurface, to help recovery by the divers from the surface (and avoid diving if possible). Note that this secondary buoy was always 3 meters underwater, to ensure navigation security.

### Field campaign

The field campaign was conducted during 4.2 months, from 14 December 2023 to 25 April 2024, in Blancs Sablons bay, north of Le Conquet, France (Fig. 1a). The site is close to Blancs Sablons, a 2.5 km long sandy beach, and is protected by the French Coastal Protection Agency (site classed Natura 2000^33^). The seabed of the bay is mainly sandy, but also rocky at some places, with the presence of kelp forests. During the winter, the site is fully exposed to the strong westerly winds, but the waves are largely dampened by the presence of many islands around the site (see the marine chart Fig. 5a). The bathymetry of the site ranges from 0 to around 15 m. The tide is semi-diurnal, with quite large range, the mean sea level reaching 4.01 m (above Chart Datum), and extreme high waters reaching 7.75 m^34^ (still above Chart Datum). A permanent tide gauge is located at Le Conquet, close to the site (1.5 km), and a permanent wave buoy is located at Pierres Noires, quite far away from the site (15 km, Fig. 5a).

A total of 30 low-cost sensors configured at 2 Hz were deployed on the Blancs Sablons site, as well as a commercial RBR solo, used as reference (Fig. 1a). The sensors were placed from 200 m to 2 km offshore. The distance between sensors was around 250 m. The position of the sensors depended on many constraints, which explains the absence of sensors at the north-east of the site (Fig. 1b). It was forbidden to place any instrument in this area, as the high seas emergency tow vessel Abeille Bourbon anchors at this place in case of stormy weather during the winter. In addition, it was essential to place the structures as far as possible from seagrass beds, which are present in some areas and protected by the Natura 2000^33^ regulations. For security reasons, and following a decision from the French authorities, it was not possible to place sensors in less than 3 m of water or along the beach, as many users visit the site, even during winter (surfers, swimmers, walkers, fishermen). Still for security reasons, it was not possible to leave any buoy in surface during all the winter, as many professional fishermen work on the site. Finally, all these restrictions show the challenges when conducting field campaigns in nearshore areas, as coastal areas combine a wide range of uses.

The deployment operation was conducted from two boats, without mobilizing divers. A small boat (Hesione 2, 6.15 m long) deployed the 10 sensors located in very shallow areas (less than 5 m depth), whereas a winch-equipped coastal boat (Albert Lucas, 11.5 m) deployed the 20 sensors located in deeper waters. All the sensors were deployed in one day (14 December 2025). The operation mobilized 9 persons.

The recovery operation was conducted from three boats, with the help of divers. On a first small boat (still Hesione 2), the divers recovered the sensors located in the shallow waters, thanks to a diving parachute (Fig. 1c). On a second small boat (Octopus, 5.30 m), the divers had to fix a surface buoy to the subsurface buoy of each deep-water structure. The winch-equipped coastal boat (still Albert Lucas) was then ready to recover the material, located thanks to the surface buoy. All the sensors were successfully recovered in three days (22, 23 and 25 April 2025). The operation mobilized 13 persons, among them 7 divers.

### Data processing to obtain water levels

The steps to convert the 2 Hz raw pressure data to water levels are the following:

- Step 1: Convert pressure data into water surface elevation. We applied the method of Karimpour and Chen^25^, using their Matlab OCEANLYZ toolbox. The process relies on the spectral method (Fast Fourier Transform). The correction for the attenuation of the dynamic pressure of the waves with depth is based on linear wave theory. The dynamic pressure to surface elevation conversion factor is computed automatically^25^. As the process results in some unrealistic amplifications, we removed the spurious peaks (i.e., water levels greater than 4 times the standard deviation, computed every 5 minutes).
- Step 2: Correct the water levels from the sensors time drift, which reaches 5.2  ± 0.4 seconds/day, depending on the sensor (Table 1, column 8). Each sensor time drift is easily computed, as the “sensor” time as well as the “computer” time (i.e., real time) are both registered in the header of the raw data pressure file, when the sensor is stopped. Note that this information was not available for the sensors which recorded only partial data (M18, M25, M29, M30) and also at M15 (for unexplained reason). In this case, we used the average time drift value (5.2 seconds/day).

**Table 1:** Characteristics of 2 Hz sea level records: sensor name, longitude, latitude, length of the record, start date, end date, missing data, time drift of the sensor, position of the Chart Datum related to the zero of the sensor.

Sensor name	Longitude	Latitude	Length of the record	Start date	End date	Missing data	Time drift	Chart Datum
M1	−4.788762	48.371257	4.2 months	2023-12-15	2024-04-20	9 h on 2024-01-27	6.38 s/day	6.52 m
M2	−4.791100	48.372748	4.2 months	2023-12-15	2024-04-20	9 h on 2024-01-27	4.81 s/day	13.48 m
M3	−4.777558	48.366530	4.2 months	2023-12-15	2024-04-20	9 h on 2024-01-27	5.18 s/day	4.79 m
M4	−4.779358	48.367781	—	—	—	—	—	—
M5	−4.781227	48.368962	4.2 months	2023-12-15	2024-04-20	9 h on 2024-01-27	5.50 s/day	10.17 m
M6	−4.782873	48.370027	4.2 months	2023-12-15	2024-04-20	9 h on 2024-01-27	5.19 s/day	10.79 m
M7	−4.784821	48.371285	4.2 months	2023-12-15	2024-04-20	9 h on 2024-01-27	5.14 s/day	13.53 m
M8	−4.786731	48.372558	4.2 months	2023-12-15	2024-04-20	9 h on 2024-01-27	5.51 s/day	13.87 m
M9	−4.788935	48.374125	4.2 months	2023-12-15	2024-04-20	9 h on 2024-01-27	5.42 s/day	13.93 m
M10	−4.774630	48.367536	4.2 months	2023-12-15	2024-04-20	9 h on 2024-01-27	4.56 s/day	5.88 m
M11	−4.777350	48.369238	2.8 months	2023-12-15	2024-03-10	9 h on 2024-01-27	5.14 s/day	11.07 m
M12	−4.779062	48.370539	4.2 months	2023-12-15	2024-04-20	9 h on 2024-01-27	5.01 s/day	12.23 m
M13	−4.780708	48.371738	4.2 months	2023-12-15	2024-04-20	9 h on 2024-01-27	4.96 s/day	13.34 m
M14	−4.782526	48.372949	4.2 months	2023-12-15	2024-04-20	9 h on 2024-01-27	4.62 s/day	15.38 m
M15	−4.784555	48.374316	4.2 months	2023-12-15	2024-04-20	9 h on 2024-01-27	—	14.16 m
M16	−4.786597	48.375760	4.2 months	2023-12-15	2024-04-20	9 h on 2024-01-27	5.41 s/day	12.81 m
M17	−4.770315	48.369650	4.2 months	2023-12-15	2024-04-20	9 h on 2024-01-27	4.97 s/day	3.40 m
M18	−4.774089	48.371528	0.6 month	2023-12-15	2024-01-02	9 h on 2024-01-27	—	11.20 m
M19	−4.775988	48.372691	4.2 months	2023-12-15	2024-04-20	9 h on 2024-01-27	4.89 s/day	12.90 m
M20	−4.777429	48.373940	4.2 months	2023-12-15	2024-04-20	9 h on 2024-01-27	5.39 s/day	15.56 m
M21	−4.779279	48.375358	4.2 months	2023-12-15	2024-04-20	9 h on 2024-01-27	5.60 s/day	16.88 m
M22	−4.781065	48.376685	4.2 months	2023-12-15	2024-04-20	9 h on 2024-01-27	5.28 s/day	17.16 m
M23	−4.782700	48.378284	4.2 months	2023-12-15	2024-04-20	9 h on 2024-01-27	5.28 s/day	16.95 m
M24	−4.768583	48.371515	—	—	—	—	—	—
M25	−4.771406	48.373698	0.1 month	2023-12-15	2023-12-18	9 h on 2024-01-27	—	10.52 m
M26	−4.766331	48.374096	4.2 months	2023-12-15	2024-04-20	9 h on 2024-01-27	5.42 s/day	3.50 m
M27	−4.765119	48.375989	4.2 months	2023-12-15	2024-04-20	9 h on 2024-01-27	5.56 s/day	3.23 m
						8 h on 2024-01-22		
						7 h on 2024-02-18		
M28	−4.764209	48.377510	—	—	—	—	—	—
M29	−4.773376	48.382138	2.7 months	2023-12-15	2024-03-07	9 h on 2024-01-27	—	9.98 m
M30	−4.769750	48.379331	3.8 months	2023-12-15	2024-04-07	9 h on 2024-01-27	—	—
- Step 3: Compute the hourly sea levels from the 2 Hz sea levels, averaging the data over 1 minute every hour. Hourly sea levels are needed further to estimate the position of the Chart Datum (step 5).
- Step 4: Correct the water levels (2 Hz and hourly) from the inverse barometer, as the bottom sensors do not record the atmospheric pressure effects. We applied the classical formulation *Δh* = −*ΔP*/(*ρg*) where *ρ* is the water density and g the acceleration due to gravity^35^. The atmospheric pressure data were linearly interpolated from three-hourly measurements at Brest-Guipavas station, located 30 km away.
- Step 5: Relate the water levels (2 Hz and hourly) to Chart Datum, removing the difference between the Chart Datum and the zero of the sensor (Fig. 2). This difference is obtained by the classical low waters concordance method^29,30^, which consists of relating the low waters at the sensor with the low waters at a reference tide gauge (here, Le Conquet) related to Chart Datum. Note that the low waters were computed from the hourly waters (obtained step 4), by fitting a polynomial of degree 2 around the low waters (2 hours before and after). Figure 2 gives an illustration of the concordance method, the difference between the Chart Datum and the zero of the sensor being the y-intercept at origin (for example, 4.79 m for M3 see Fig. 2a). The Chart Datum values for all the sensors are given Table 1.

**Fig. 2:**
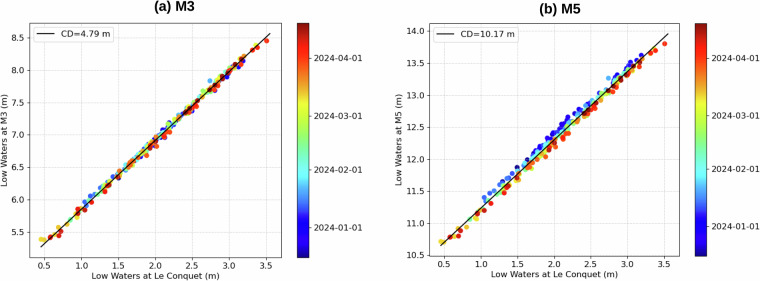
(**a**) Estimation of the difference between the Chart Datum and the zero of the sensor by the classical low waters concordance method at (**a**) M3 and (**b**) M5. The y-intercept at origin gives the position of the Chart Datum related to the zero of the sensor (see the value in the legend at the top left corner). Note that low waters at Le Conquet are related to Chart Datum, whereas low waters at the sensor are related to the zero of the sensor.

**Fig. 3:**
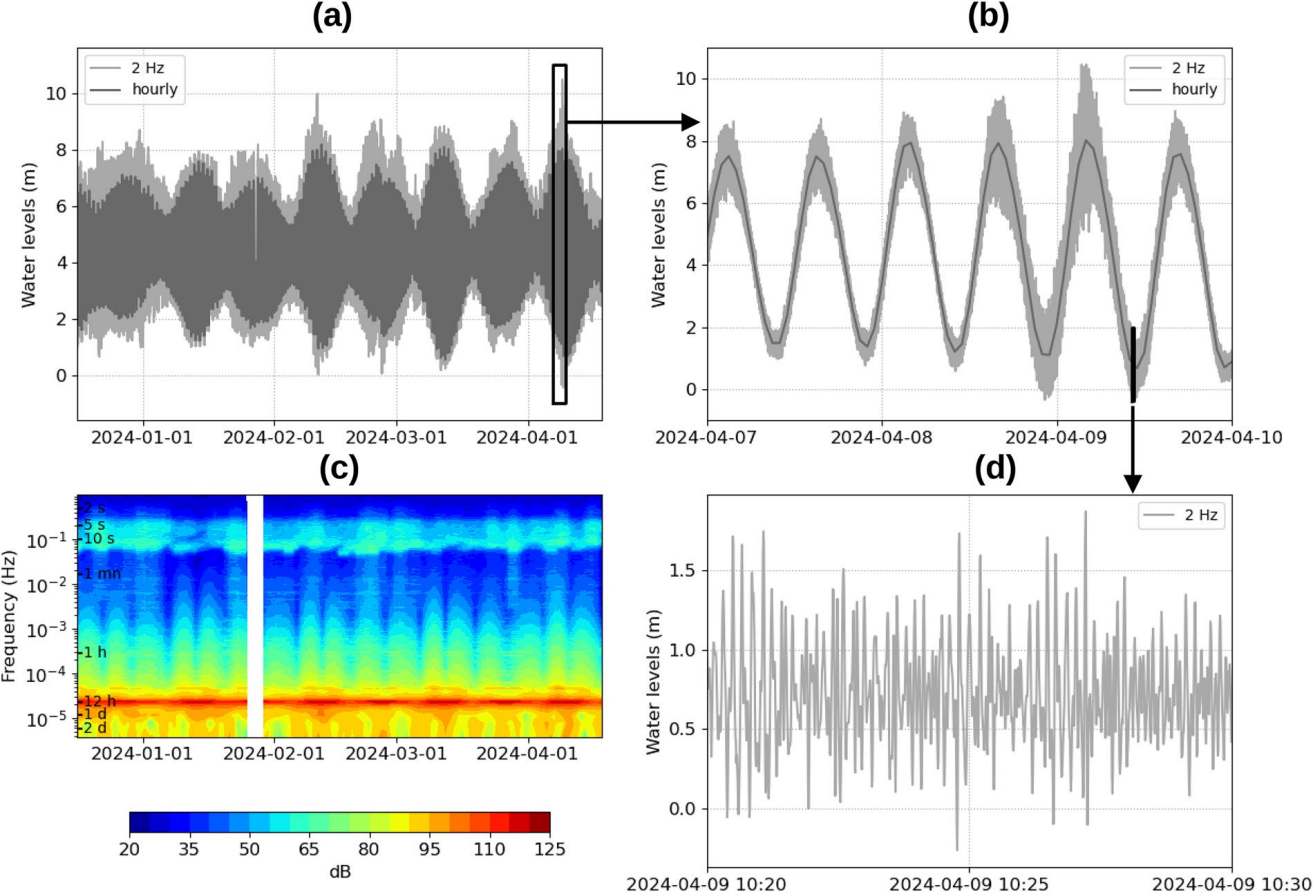
Water levels at M1 during (**a**) 4 months (**b**) 3 days (**d**) 10 minutes. (**d**) Spectrogram of 2 Hz water levels at C1. Spectra is computed every day on a 3-day sliding window.

We finally obtain the water levels at 2 Hz related to Chart Datum (Fig. 3a,b,d). These data are available for the scientific community^31^ (see Data Records). The spectrogram of the 2 Hz water levels is computed at M1, based on daily spectra over a 3-day sliding window (Fig. 3c). This spectrogram reveals the signature of the tide (see the red line at around 12h25), and the waves (see the light blue line around 5 to 10 s).

### Computation of waves and surges

The Significant Wave Height is computed hourly, as four times the standard deviation of the (2Hz) water levels^36^, selected on a 5 minute window around each hour.

The surges are computed hourly, removing the tide and mean sea level from the hourly water levels. Tide and mean sea level are computed over all the period of measurements (around 4 months), using Utide tidal analysis software^37^. However, with only 4 months of data, the tide is not precise enough (due to shallow water distortion of the tide, 1 year would be more appropriate^30^), and the residual still displays large oscillations at tidal frequency, even after tidal removal. These oscillations are removed applying a 4th order low-pass Butterworth filter, with cutoff frequency 16 hours^38^. Note that no trend is applied in Utide (trend=False, whereas True is applied by default). Finally, note that we did not compute the tide and surges, for records shorter than 1 month (M18 and M25), due to high imprecision of tide with such short records.

The surges at Le Conquet permanent tide gauge (hourly data since 1970) are estimated by removing the tide and the mean sea level, computed over the 20 last years (2004–2024, still using Utide). Note that the sea level trend is removed, to get rid of sea level rise over the 20 years (trend=True in Utide). At Le Conquet, no Butterworth filter is applied, as 20 years is long enough to estimate robustly the tide.

## Data Records

The dataset is available in SEANOE (Sea Scientific Open Data Publication) repository^31^ (10.17882/105438). The dataset includes raw pressure data and sea level data at 2 Hz at the 27 positions, as well as a coordinate file (text format), with longitude and latitude of the sensors. A ’readme’ file describes the repository.

### Raw pressure data

The raw pressure data consists of 27 files in text format (csv). Data are sampled at 2 Hz, and recorded from December 2023 when the sensors were configured, to April 2024, when they were stopped. The pressure files (climex_blancs_sablons_M*_P.csv) contain a header (10 lines), and then one line per time record (every 0.5 s), with the date, the temperature (^∘^C), and the pressure (bar). Each file size is 1.3 Go (when complete). Note that raw pressure data are not corrected for the time drift of the sensors (Table 1).

### Sea level data

The sea level data consist of 27 files in NetCdf format (climex_blancs_sablons_M*_sea_level.nc). Data are sampled at 2 Hz and were processed following steps 1–5 described in the Methods section. The ‘sea_level’ variable is expressed in meters and related to Chart Datum. The ‘time’ variable is expressed in seconds and represents the number of elapsed days since 1970-01-01. Each file size is 335 Mo (when complete). The coordinates, length record, start date, end date, and missing data for each sensor are summarized Table 1.

## Technical Validation

### General performance

Among the 30 low-cost sensors, 22 sensors functioned properly, recording full data at 2 Hz over 4.2 months (blue dots on Fig. 1b). Five sensors recorded only partial data (green dots on Fig. 1b), due to battery failure (M18, M25, M29, M30) or malfunctioning (M11, which recorded a wrong constant pressure from 10 March 2024). Three sensors did not work (M4, M24, M26, see red dots on Fig. 1b), due to holes in their protecting plastic bags, possibly caused by small animals such as juvenile crabs (young crabs of a few mm were found in the PVC protecting tubes).

There are only few gaps in data (Table 1, column 5): 9 hours on 2024-01-27 for all the sensors, 8 hours on 2024-01-22 and 7 hours on 2024-02-18 for sensor M27. Note that very small gaps (from 0.5 to 4 seconds) may occur in the water heights records (but not in raw pressure data). These gaps are negligible compared to the total length of the record (one hour maximum cumulating all the gaps, compared to 4.2 months of total record).

### Comparison of pressure with the RBR reference sensor

**Fig. 4:**
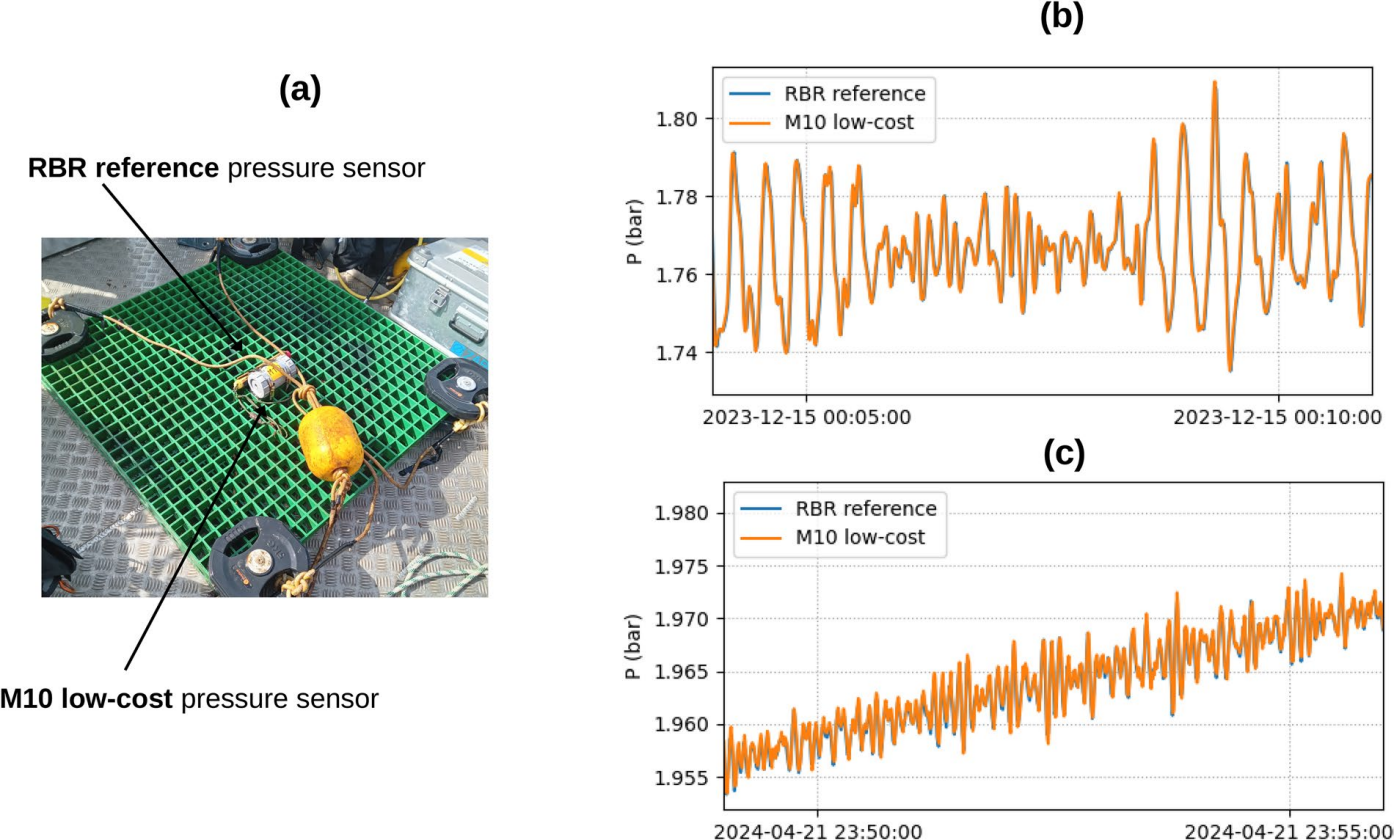
(**a**) Structure with RBR reference pressure sensor and M10 low-cost pressure sensor fixed together (**b**) Pressure at both M10 and RBR sensors during 7 minutes at the beginning of the experiment (2023-12-15) (**c**) Same as (**d**) but at the end of the experiment (2024-04-21).

We compared the pressure from the M10 low-cost sensor with the RBR solo reference instrument (Fig. 4a), both instruments were fixed together on the M10 structure. Note that the RBR samples at 1 Hz, with an accuracy of 2.5 cm (i.e.,  ±0.05% of the 50 m full scale).

The M10 pressure was corrected from the theoretical sensor time drift (4.56 s/day, Table 1), estimated from the information in the header of the pressure file (see step 2 in Methods section), and from an additional small time drift (33 seconds over the the whole period of 4.2 months), revealed by the comparison between the two sensors. Note that this additional time drift is practically negligible, except for wave-to-wave comparison between sensors.

The M10 pressure was also corrected from a daily offset, revealed by the comparison between the two sensors (Fig. 6b). The mean offset reaches 21 mbar, whereas the daily offset varies between  ± 5 mbar around the mean offset. The mean offset (21 mbar) has no impact on the quality of measurements, as the vertical reference is arbitrary. But the non-stationary daily offset reveals low frequency differences between the two instruments, which may reach a few cm (1 mbar being equivalent to  −1 cm). The linear trend of the daily offset (obtained fitting a linear regression on the whole dataset, see Fig. 6b) reaches  −1 mbar/month, resulting in spurious trends in water levels of +1 cm/month at M10 (see the Usage notes for more details). Note that these changes, expressed in cm/month, are not linear (Fig. 6b).

Once the pressure at M10 has been corrected from the time drift and the daily offset (i.e., the low-cost sensor daily mean is set equal to the RBR daily mean), the pressure at both M10 and RBR sensors match very well over the whole record, as shown Fig. 4b,c. The wave-to-wave comparison is very good, from the beginning fo the experiment (2023-12-15, Fig. 4b), until the end of the experiment (2024-04-21, Fig. 4c). The Root Mean Square Error between the two instruments is 0.009 bar over the whole period.

### Comparison of the surges with permanent tide gauge at Le Conquet

**Fig. 5:**
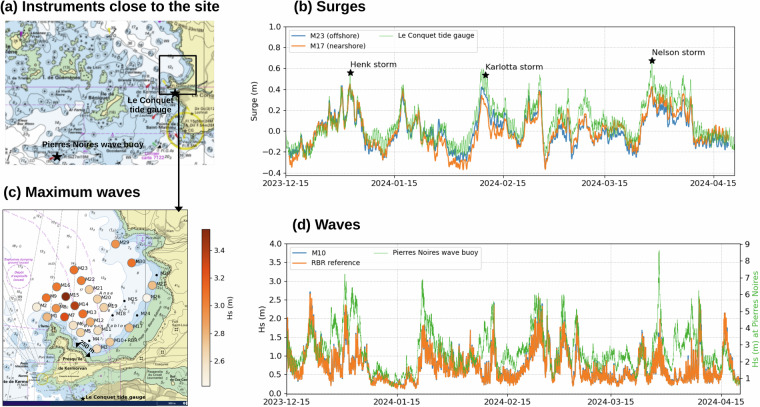
(**a**) Location of the permanent instruments (Le Conquet tide gauge and Pierres Noires wave buoy) close to the site (**b**) Surges at M23, M17 and Le Conquet tide gauge during the experiment (**c**) Maximum Significant Wave Height during the field campaign (4.2 months) (**d**) Significant wave height at M10, RBR and Pierres Noires wave buoy during the experiment.

We computed the surges at all the sensors, for comparison with the closest permanent tide gauge, located at Le Conquet (see the location of the tide gauge on Fig. 5a), and operated by French Hydrographic Office since 1970. As shown Fig. 5b for sensors M17 and M23, the surges match quite well with Le Conquet, despite the tide is estimated using different periods at sensors (maximum of 4.2 months) and Le Conquet (20 years). This underlines that the surges can be well estimated on short periods, applying a 4th order Butterworth filter after removing the tide (see Methods section). The Root Mean Square Error between the surges at M23 and Le Conquet is 0.11 m, the bias is  −0.08 m. Similar values are found between M17 and Le Conquet. The largest surges (around 0.5 m) reveal the storms that hit the site during the experiment (Fig. 5b), such as Henk storm (2 January 2024), Karlotta storm (10 February 2024) or Nelson storm, which occurred unusually late in the storm surge season (28 March 2024).

### Comparison of the waves with the RBR reference sensor and the permanent wave gauge at Pierres Noires

We computed the significant wave height at all the sensors, for comparison with the RBR reference sensor and with the closest permanent wave buoy, located at Pierres Noires (see the location of the wave buoy on Fig. 5a). The Pierres Noires wave gauge is operated by CEREMA (Centre for Studies on Risks, the Environment, Mobility and Urban Planning) since 2005.

The Fig. 5d shows that the significant wave height match very well between the M10 low-cost sensor and the RBR reference sensor (Root Mean Square Error of only 4 cm), which is not surprising as both sensors measure similar bottom pressure (see Fig. 4). In addition, the significant wave height at M10 and RBR match quite well with Pierres Noires wave buoy, despite different amplitudes (see the different y-axis, significant wave height reaching 3 m on site, against 9 m at Pierres Noires). Indeed, the direct comparison of amplitudes is not possible, as the waves are really smaller on Blancs Sablons site compared to Pierres Noires, the Blancs Sablons site being protected by the presence of many islands (see the marine chart Fig. 5a).

In the Blancs Sablons bay, the maximum significant wave height over the whole period ranges from 2.4 to more than 3.5 m, the highest values being obtained offshore (3.55 m at M15, Fig. 5c).

## Usage Notes

**Fig. 6:**
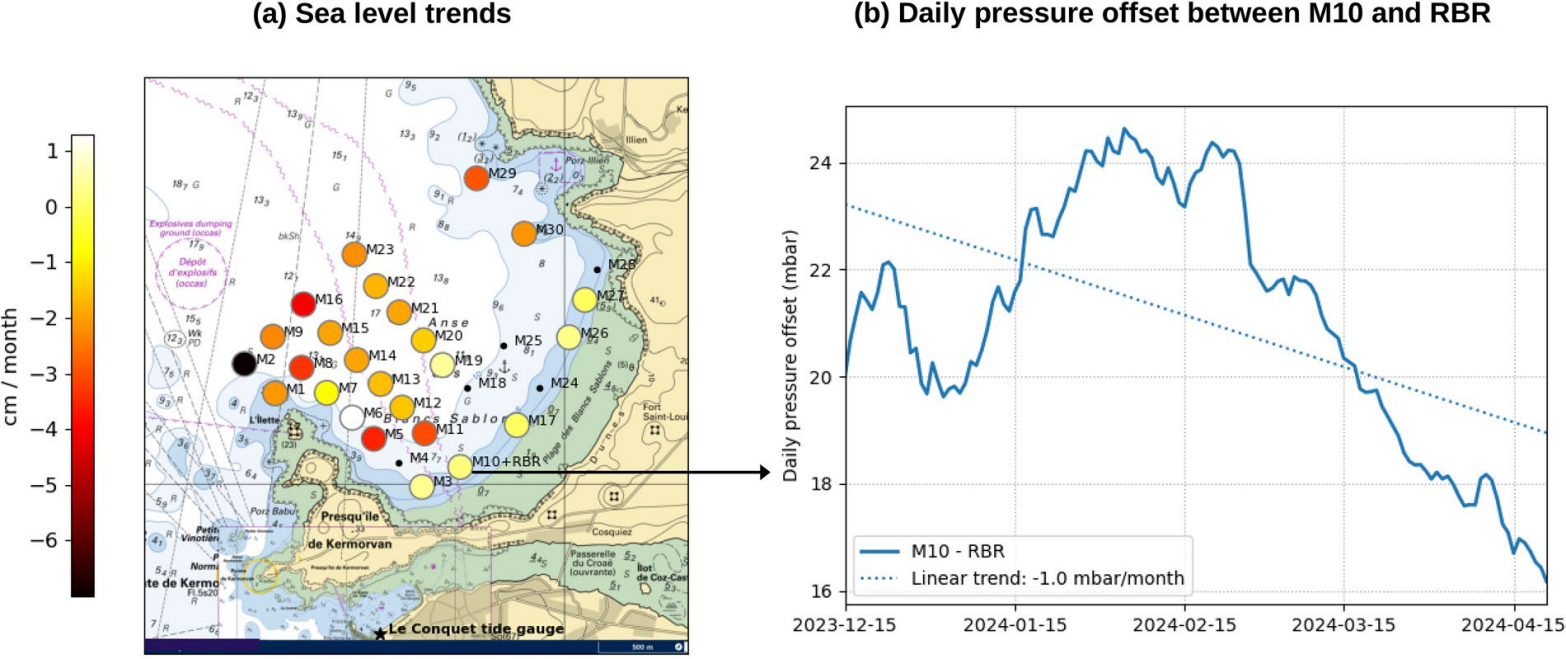
(**a**) Sea level linear trends (expressed in cm/month) (**b**) Daily bottom pressure offset between low-cost M10 sensor and RBR reference sensor.

We computed the sea level trends (expressed in cm/month), fitting a linear regression on hourly sea level data over the whole record (Fig. 6a). Overall, sea level data display negative trends, reaching  −1.7 cm/month in average. These trends vary from +1 cm/month at M6 to  −7 cm/month at M2. Such trends can be partly due to sensor drifting (e.g., 1 cm/month reported at M10, Fig. 6b) or vertical movement (e.g., instrument’s sinking into the sand). Whatever the causes, these significant trends lead to consider the data with caution for applications requiring a very stable vertical reference, for example, when computing the wave setup (an additional surge due to wave dissipation in nearshore areas^14^), or when investigating long-term trends (several decades being generally required).

At M10, the sea level trend is close to zero (0.2 cm/month, see Fig. 6a), whereas a small drift of 1 cm/month was previously reported (see the Technical validation section and Fig. 6b). The discrepancies between the instrument spurious drift (1 cm/month) and the sea level drift (0.2 cm/month) suggests that at M10, the structure possibly moved vertically, at a small rate of around  −0.8 cm per month.

Finally, note that at the end of the experiment, two sensors were found buried under approximately 50 cm of sand (M27 and M28, located at the north end of the beach). M28 sensor did not work, but M27 sensor successfully recorded data during all the experiment. The absence of sea level trend at this position (−0.05 cm/month at M27) suggests that the structure was vertically stable, and did not sink (neglecting possible spurious drift from the sensor). More likely, the structure has been covered by the sand around, which was transported by the wave action.

## Data Availability

The Ocean Wave Analyzing Toolbox OCEANLYZ^25^, to convert pressure data into water surface elevation data, is available at https://github.com/akarimp/Oceanlyz and documented at https://oceanlyz.readthedocs.io/en/latest/index.html. The Python Utide tidal analysis software^37^ is available on the Utide website, https://pypi.org/project/UTide/.
